# The Edible Fungi

**Published:** 1883-07

**Authors:** 


### THE EDIBLE FUNGI.

IN spite of occasional fatal accidents through the inadvertent eating of poisonous species, fungi are largely consumed, both by savage and civilized men in all parts of the world. Among the Fuegians, certain kinds form, with shell fish, their staple food. The natives of Australia use largely a truffle, which attains a weight of more than two pounds, and is known under the name of native bread. The Chinese, who are singularly free from prejudice in the matter of food, are especially fond of them, and for some years past New Zealand has exported large quantities of an edible fungus to San Francisco and Hong Kong for the use of Celestials. Consul Griffin, of Auckland, in a recent report, gives a full account of this new industry, the gathering and drying of the fungus giving profitable employment to large numbers of colonial children, as well as of Maories. The species grows abundantly in the wooded regions of New Zealand, and, when dry, is worth from two to five pence per pound.

The Chinese use it, as they do the edible swallow's nest, as a chief ingredient in their favorite soup. They also employ it as a medicine, and, stranger still, for making a valuable dye for silk. Another remarkable edible fungus of New Zealand, is the *Sphœria Robertsii*, which grows out of the body of a large caterpillar, practically converting the latter into a vegetable substance. The caterpillar lives underground, and the fungus springs upward through the soil till it reaches a height of eight or ten inches. It is eaten by the Maories, who employ it also, when burned, as a coloring matter. Among the northeastern tribes of Asia, fungi are largely used as food. One species, when pounded, forms their snuff, while another —the Fly Agaric—which is utilized in Europe as a fly-killer, and is regarded as one of the most poisonous forms, is used by them as a substitute for ardent spirits, the eating of a single large specimen being sufficient "to produce a pleasant intoxication for a whole day." In many parts of Europe fungi are a favorite food, being eaten fresh, and also preserved in vinegar for winter use. For pickling purposes, all kinds, it is said, are gathered, the vinegar being said to neutralize the alkaline posion of the noxious species. The common mushroom, the morel, and the truffle are, however, the favorite edible fungi.

